# Using deep leaning models to detect ophthalmic diseases: A comparative study

**DOI:** 10.3389/fmed.2023.1115032

**Published:** 2023-03-01

**Authors:** Zhixi Li, Xinxing Guo, Jian Zhang, Xing Liu, Robert Chang, Mingguang He

**Affiliations:** ^1^State Key Laboratory of Ophthalmology, Zhongshan Ophthalmic Center, Sun Yat-Sen University, Guangdong Provincial Key Laboratory of Ophthalmology and Visual Science, Guangdong Provincial Clinical Research Center for Ocular Diseases, Guangzhou, China; ^2^Wilmer Eye Institute, Johns Hopkins University, Baltimore, MD, United States; ^3^Department of Ophthalmology, Byers Eye Institute at Stanford University, Palo Alto, CA, United States

**Keywords:** deep learning, diabetic retinopathy, age-related macular degeneration, glaucomatous optic neuropathy, fundus photograph

## Abstract

**Purpose:**

The aim of this study was to prospectively quantify the level of agreement among the deep learning system, non-physician graders, and general ophthalmologists with different levels of clinical experience in detecting referable diabetic retinopathy, age-related macular degeneration, and glaucomatous optic neuropathy.

**Methods:**

Deep learning systems for diabetic retinopathy, age-related macular degeneration, and glaucomatous optic neuropathy classification, with accuracy proven through internal and external validation, were established using 210,473 fundus photographs. Five trained non-physician graders and 47 general ophthalmologists from China were chosen randomly and included in the analysis. A test set of 300 fundus photographs were randomly identified from an independent dataset of 42,388 gradable images. The grading outcomes of five retinal and five glaucoma specialists were used as the reference standard that was considered achieved when ≥50% of gradings were consistent among the included specialists. The area under receiver operator characteristic curve of different groups in relation to the reference standard was used to compare agreement for referable diabetic retinopathy, age-related macular degeneration, and glaucomatous optic neuropathy.

**Results:**

The test set included 45 images (15.0%) with referable diabetic retinopathy, 46 (15.3%) with age-related macular degeneration, 46 (15.3%) with glaucomatous optic neuropathy, and 163 (55.4%) without these diseases. The area under receiver operator characteristic curve for non-physician graders, ophthalmologists with 3–5 years of clinical practice, ophthalmologists with 5–10 years of clinical practice, ophthalmologists with >10 years of clinical practice, and the deep learning system for referable diabetic retinopathy were 0.984, 0.964, 0.965, 0.954, and 0.990 (*p* = 0.415), respectively. The results for referable age-related macular degeneration were 0.912, 0.933, 0.946, 0.958, and 0.945, respectively, (*p* = 0.145), and 0.675, 0.862, 0.894, 0.976, and 0.994 for referable glaucomatous optic neuropathy, respectively (*p* < 0.001).

**Conclusion:**

The findings of this study suggest that the accuracy of this deep learning system is comparable to that of trained non-physician graders and general ophthalmologists for referable diabetic retinopathy and age-related macular degeneration, but the deep learning system performance is better than that of trained non-physician graders for the detection of referable glaucomatous optic neuropathy.

## Introduction

Diabetic retinopathy (DR), glaucomatous optic neuropathy (GON), and age-related macular degeneration (AMD) are responsible for more than 18% of visual impairment and blindness cases globally (1–6). While it is estimated that 80% of vision loss is avoidable through early detection and intervention (7–9), approximately 50% of cases remain undiagnosed (10, 11). High rates of undiagnosed disease can be attributed to these conditions being asymptomatic in their early stages, coupled with a disproportionately low availability of eye care services, particularly within developing countries and under-served populations (12).

Previous research has demonstrated that color fundus photography is an effective tool for the diagnosis of AMD, GON, and DR (13–15). Despite this, accurate interpretation of the optic nerve and retina is highly dependent on clinical experts, limiting the utility in low recourse settings. Deep learning represents an advancement of artificial neural networks that permits improved predictions from raw image data (16). Recently, several studies have investigated the application of deep learning algorithms for the automated classification of common ophthalmic disorders (17–21), with promising results for disease classification (sensitivity and specificity range = 80–95%). Thereby, these systems offer great promise to improve the accessibility and cost-effectiveness of ocular disease screening in developing countries.

Despite this, most previous systems could only detect a single ocular disorder, thus would omit severe blinding eye diseases. In addition, previous studies have evaluated on retrospective datasets, and there is a paucity of data directly comparing the performance of deep learning system (DLS) capable to detect common blindness diseases to that of general ophthalmologists or non-physician graders. Given the fact that in real world screening programs, human graders or general ophthalmologists may also make mistakes, a robust study to directly compare DLS and general ophthalmologists or non-physician graders is of paramount importance for healthcare decision makers and patients to make informed decisions relating to the deployment of these systems.

Therefore, in the present study, we investigated the diagnostic agreement between ophthalmologists with varying levels of experience, non-physician graders, and validated deep learning models (22) for DR, GON, and AMD on an independent dataset in China.

## Methods

This study was approved by the Institutional Review Board of the Zhongshan Ophthalmic Center, China (2017KYPJ049) and conducted in accordance with the Declaration of Helsinki. All graders and ophthalmologists have been informed that their data will be compared with the DLS. Informed consent for the use of fundus photographs was not required as images were acquired retrospectively and were fully anonymized.

### Test set development, reference standard, and definitions

A total of 300 fundus photographs were randomly selected from a subset of 42,388 independent gradable images from the online LabelMe dataset (http://www.labelme.org, Guangzhou, China) (22, 23). The LabelMe dataset includes images from 36 hospital ophthalmology departments, optometry clinics, and screening settings in China that include various kinds of eye diseases, such as DR, glaucoma, and AMD. The data will be available upon request. Retinal photographs were captured using a variety of common conventional desktop retinal cameras, including Topcon, Canon, Heidelberg, and Digital Retinography System. The LabelMe dataset was graded for DR, GON, and AMD by 21 ophthalmologists who previously achieved an unweighted kappa of ≥0.70 (substantial) on a test set of images. Images were randomly assigned to a single ophthalmologist for grading and were returned to the pooled dataset until three consistent grading outcomes were achieved. Once an image was given a reference standard label it was removed from the grading dataset. This process has been described in detail elsewhere (22, 23).

Stratified random sampling was used to select 50 images of each disease category and an additional 150 images classified as normal or a disease other than DR, AMD, and GON. Poor quality images (defined as ≥50% of the fundus photograph area obscured) were excluded. Images that were included in the training and internal validation datasets of the deep learning models were not eligible for inclusion. Following the selection of images, experienced retinal (*n* = 5) specialists independently labeled all 300 images to establish a reference standard for DR and AMD. Similarly, glaucoma specialists (*n* = 5) independently graded all images to determine the GON reference standard. Specialists were blinded to any previous medical history or retinal diagnosis for the included images. Once all images were graded, they were converted to a two-level classification for each disease: non-referable and referable. Each image was only assigned a conclusive label if more than 50% of the specialists reported a consistent grading outcome.

A website^1^ was developed to allow human graders to log in and interpret images. Diabetic retinopathy severity was classified as none, mild non-proliferative DR (NPDR), moderate NPDR, severe NPDR, and proliferative DR using the International Clinical Diabetic Retinopathy scale (24). Diabetic macular edema (DME) was defined as any hard exudates within one-disk diameter of the fovea or an area of hard exudates in the macular area at least 50% of the disk area (25). Referable DR was defined as moderate NPDR or worse with or without the presence of DME. The severity of AMD was graded according to the clinical classification of AMD, which has been described elsewhere (26). For the purpose of this study, referable AMD was defined as late wet AMD as it was the only subtype of AMD that could be managed with effective therapy currently. Glaucomatous optic neuropathy was classified as absent or referable GON according to definitions utilized by previous population-based studies (27–29). The definition of referable GON included the presence of any of the following: vertical cup to disk ratio (VCDR) ≥0.7; rim width ≤0.1 disk diameter; localized notches; and presence of retinal nerve fiber layer (RNFL) defect and/or disk hemorrhage.

### Development of the deep learning system

The development and validation of the DR, GON, and AMD models have been described in detail elsewhere (22, 30–32). In brief, referable GON, DR, and AMD deep learning algorithms were developed using a total of 210,473 fundus photographs (referable DR, 106,244; referable GON, 48,116; referable AMD 56,113). Several pre-processing steps were performed for normalization to control for variations in image size and resolution. This included augmentation to enlarge heterogeneity, applying local space average color for color constancy and downsizing image resolution to 299 × 299 pixels (33). Finally, eight convolutional neural networks were contained within the DLS (Version 20,171,024), all adopting Inception-v3 architecture (34). The development of the networks was described in our previous studies (22, 23, 32). Briefly, the networks were downsized to 299 × 299, and local space average color and data augmentation were adopted. These networks were trained from scratch and included (1) classification for referable DR, (2) classification of DME, (3) classification of AMD, (4) classification of GON, and (5) assessment of the availability of the macular region and rejection of non-retinal photographs.

### Graders and ophthalmologists identification and recruitment

Five trained non-physician graders, who also previously received training for DR, AMD, and GON classification, usually graded images from 50 to 100 participants for common blindness diseases every workday and underwent tests per quarter, from Zhongshan Ophthalmic Center Image Grading Center with National Health Screening (NHS) DR grader certification were recruited to grade all these images.

We also invited general ophthalmologists from four provincial hospitals and five county hospitals in seven provinces in China (Guangdong, Guangxi, Fujian, Jiang Su, Yunnan, Xinjiang, and Inner Mongolia province). General ophthalmologists who had at least 3 years clinical practice including residency were eligible to participate.

Selected ophthalmologists were sent an invitation to participate *via* email or mobile phone text message. Those who did not respond were followed up with a telephone call. The clinical practice characteristics of invited ophthalmologists were obtained from publicly available resources or personally *via* telephone.

**Figure 1:**
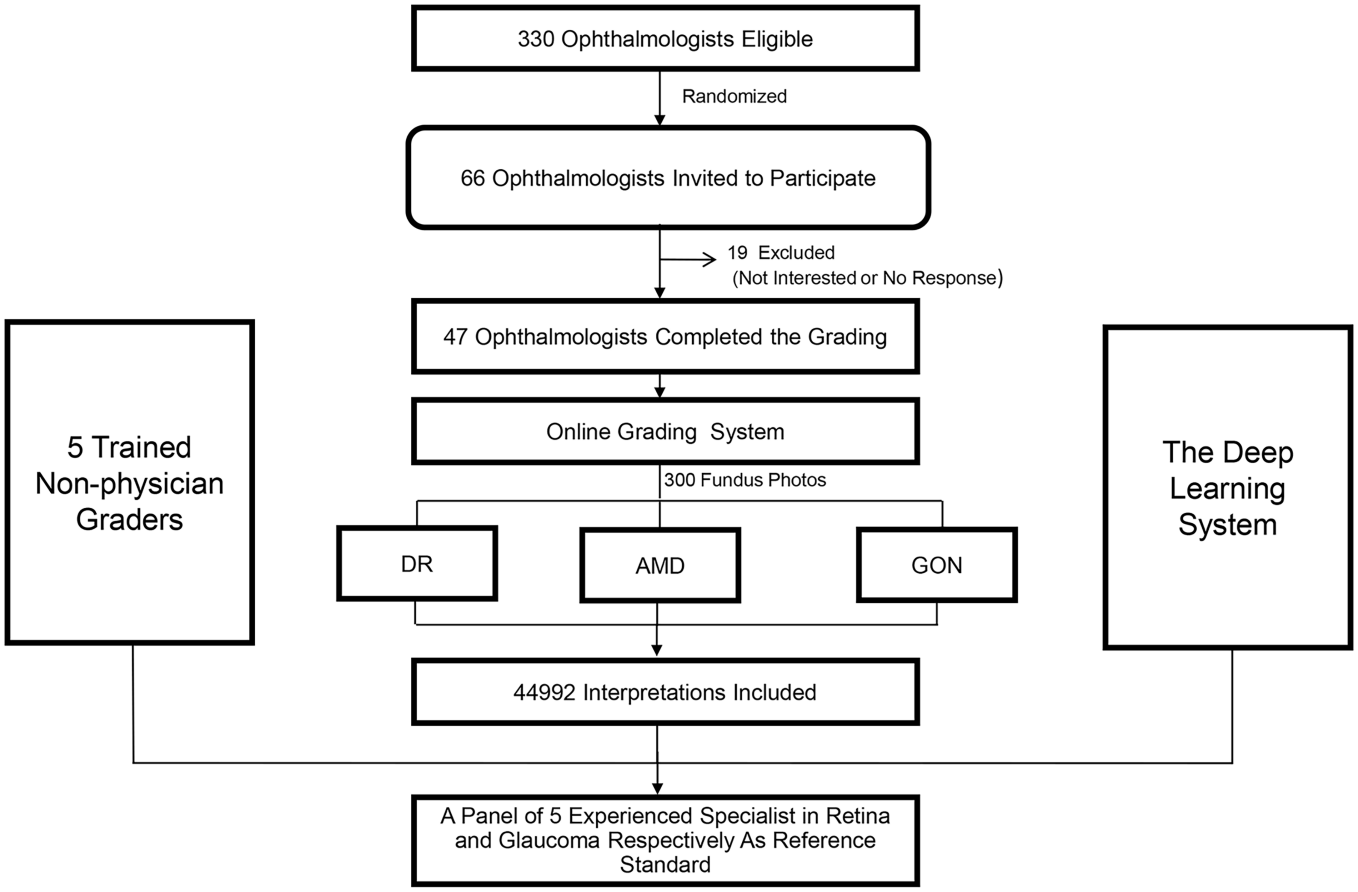
Recruitment, workflow, and grading of ophthalmologists and non-physician graders.

Of the 330 ophthalmologists who were eligible to participate, 66 (20%) were randomly selected and subsequently invited to participate in the study. Nineteen ophthalmologists (28.8%) declined or did not respond and 47 ophthalmologists (71.2%) agreed to participate. A flow chart outlining the recruitment of ophthalmologists is shown in Figure 1.

### Test set implementation

Participants independently reviewed all 300 images in a random order. They were blinded to the reference standard and the grades assigned by other participants. Due to the variability in existing classification criteria for GON, a standardized grading criteria was provided to all participants. Participants were not provided with details of the comprehensive grading criterion utilized for the grading of DR and AMD, as it was assumed that the participants’ experience would be sufficient to enable them to classify these disorders into the specific categories (DR: mild, moderate, severe NPDR and proliferative DR; AMD: early or moderate AMD, late dry AMD, and late wet AMD). There was no time limit for the interpretation of each image. All grading results were converted to a two-level classification for each disease (referable and non-referable disorders) and then compared against the reference standard. The eight deep learning models were also tested using the same images.

In order to characterize the features of misclassified images by DLS and human graders, an experienced ophthalmologist (Z.X.L.) reviewed misclassified fundus photographs and classified them into categories arbitrarily developed by a consensus meeting by investigators.

### Statistical analysis

The area under the receiver operating characteristic curve (AUC), rate of agreement and unweighted kappa were calculated. Agreement was defined as the proportion of images that were correctly classified by participants or the DLS models using the gold standard label as a reference standard. Firstly, data from all participants were used and in this situation, the CIs accounting for within and between subject variability by estimating the variance using the form; {var.(parameter_p_) + [avg(parameter_p_) × (1−avg(parameter_p_))]/*n*_c_}/*n*_p_, where avg.(parameter_p_) denotes the average corresponding parameter (AUC, agreement rate or kappa) among participants, var.(parameter_p_) denotes the sample variance of parameter among participants, *n*_c_ denotes the number of images interpreted by each participant, and *n*_p_ denotes the number of participants.

Then, a representative grading result for graders and ophthalmologists was made when more than 50% of group members achieved consistent grading outcomes. As the DLS can generate a continuous probability between 0 and 1 for referable disorders, AUC for DLS was calculated using these continuous probabilities to compared with reference standard, whereas the agreement rate and unweighted kappa were dichotomized by assigning a certain probability when reaching the highest accuracy. The AUCs of graders, ophthalmologists, and DLS were calculated by comparing with reference standard for two-level classification (referable and non-referable).

We investigated the extent to which the clinical experience of ophthalmologists was associated with agreement. Logistic regression models of ophthalmologist agreement that simultaneously incorporated several ophthalmologist characteristics (hospital level, academic affiliation, clinical practice years, and clinical expertise) were modeled. Non-physician graders were not included in this analysis due to the relatively small sample size (*n* = 5).

Sensitivity analyses was used to explore whether the grading results would change by using an alternate reference standard instead of the specialist-derived standard. Firstly, cases where the reference standard was different from the most frequent (≥80.0%) grading result of the participants were identified (8 of 300 images). Then, the results were reanalyzed by substituting the most frequent grading outcome of participants as the reference standard for the eight images, or just excluding the eight images. A *p* value of less than 0.05 was regarded as statistically significant. Stata statistical software (version 14; College Station, Texas, United States) was used.

## Results

### Reference dataset

Of the 300 images included in the dataset, the total number of images labeled as referable DR, AMD, and GON according to the final specialist grading were 45 (15.0%), 46 (15.3%), and 46 (15.3%), respectively. The remaining 163 (54.4%) images were classified as normal or a disease other than DR, AMD, and GON.

### Graders and ophthalmologists characteristics

The five trained non-physician graders were all females with a mean age of 30.4 ± 2.2 years (range, 27–34 years) and an average of 3.6 ± 0.6 years (range, 2–5 years) of grading experience in DR screening support and research image grading. There were 6, 23, 12, and 6 general ophthalmologists aged <30, 30–40, 40–50, and ≥50 years, respectively. Among these ophthalmologists, there were 22 males and 25 females. Twenty-seven were from affiliated hospitals and the other were from nonaffiliated hospitals. Their lengths of clinical practice were 5 years (*n* = 13), 5–10 years (*n* = 16), and ≥10 years (*n* = 18).

### Diagnostic agreement among deep learning models, trained non-physician graders, and ophthalmologists

Table 1 displays the agreement distribution by individual grading outcomes of specialists performing initial reference standard grading compared to the final reference standard. The overall agreement rate of the initial independent specialist diagnoses was 96.5% for referable DR, 98.1% for referable AMD, and 92.8% for referable GON.

**Table 1:** Comparison of the five specialist ophthalmologist’s independent gradings vs. final expert consensus reference standard for 300 fundus photographs.^a^

	Specialist ophthalmologists independent gradings			
Final reference standard	Absent	Present	Missing	Total
Referable DR^b^				
Absent	1,269	6	0	1,275
Present	45	178	2	225
Total	1,314	184	2	1,500
Late wet AMD^c^				
Absent	1,258	12	0	1,270
Present	16	214	0	230
Total	1,274	226	0	1,500
Referable GON^d^				
Absent	1,176	94	0	1,270
Present	14	216	0	230
Total	1,190	310	0	1,500

^a^The overall all agreement rate for referable DR, late wet AMD, and GON were 96.5, 98.1, and 92.8%, respectively. ^b,c^The members to make reference standard were consisted of five retina specialists, and each disorder was graded for multiple categories and then converted to two levels for analysis.^d^The members were consisted of five glaucoma specialists. DR, diabetic retinopathy; AMD, age-related macular degeneration; GON, glaucomatous optic neuropathy.

Table 2 provides a comparison between the DLS and general ophthalmologists. The sensitivity and specificity of the DLS for referable DR were 97.8% (44/45) and 92.5% (236/255), respectively. The results for general ophthalmologists for referable DR were 91.1% (41/45) and 99.6% (254/255), respectively.

**Table 2:** Comparison of deep learning system and general ophthalmologists to the expert consensus reference standard.

	Reference standard	Deep learning system			Ophthalmologists		
		Agreement (%)	Misclassification (%)	Total	Agreement (%)	Misclassification (%)	Total
Diabetic	Referable	44 (97.8)	1 (2.2)	45	41 (91.1)	4 (8.9)	45
Retinopathy	Non-referable	236 (92.5)	19 (7.5)	255	254 (99.6)	1 (0.4)	255
Age related macular degeneration	Referable	39 (83.0)	8 (7.0)	47	43 (91.5)	4 (8.5)	47
	Non-referable	245 (96.8)	8 (3.2)	253	248 (98.0)	5 (2.0)	253
Glaucomatous optic neuropathy	Referable	45 (97.8)	1 (2.2)	46	42 (91.3)	4 (8.7)	46
	Non-referable	252 (99.2)	2 (0.8)	254	249 (98.0)	5 (2.0)	254

A representative grading result for graders and ophthalmologists were made when more than 50% of group members achieved a consistent grading.

Table 3 compares the grading agreement of trained non-physician graders, ophthalmologists, and the DLS versus the reference standard. There were no significant differences in the AUC of non-physician graders, general ophthalmologists with different levels of clinical experience, and the DLS for the interpretation of referable DR (*p* = 0.415, compared with expert consensus reference diagnosis) and referable AMD (*p* = 0.145, compared with expert consensus reference diagnosis). For the classification of GON, the DLS achieved a superior AUC result compared to non-physician graders (*p* < 0.001).

**Table 3:** Agreement of image interpretation by trained non-physician graders, general ophthalmologists, and deep learning system versus the expert consensus reference standard.^a^

	Trained non-physician graders (95% CI)	Ophthalmologists (95% CI)				Deep learning system^a^ (95% CI)	*p* value
		Clinical experience 3–5 years	Clinical experience 5–10 years	Clinical experience >10 years	Total		
Referable DR							
*Model 1*							
AUC	0.984 (0.960–1.000)	0.964 (0.926–1.000)	0.965 (0.927–1.000)	0.954 (0.911–0.996)	0.954 (0.911–0.995)	0.990 (0.982–0.999)	0.415
Kappa	0.959 (0.845–1.000)	0.946 (0.832–1.000)	0.947 (0.834–1.000)	0.933 (0.820–1.000)	0.933 (0.820–1.000)	0.775 (0.665–0.886)	
Agreement rate	0.989 (0.971–0.998)	0.983 (0.961–0.996)	0.987 (0.966–0.996)	0.983 (0.961–0.995)	0.983 (0.962–0.995)	0.933 (0.899–0.959)	
Referable AMD							
*Model 1*							
AUC	0.912 (0.859–0.964)	0.933 (0.887–0.979)	0.946 (0.904–0.987)	0.958 (0.922–0.995)	0.948 (0.906–0.989)	0.945 (0.903–0.986)	0.145
Kappa	0.823 (0.710–0.936)	0.851 (0.738–0.964)	0.876 (0.762–0.989)	0.901 (0.788–1.000)	0.887 (0.774–1.000)	0.798 (0.685–0.911)	
Agreement rate	0.953 (0.923–0.974)	0.960 (0.931–0.979)	0.967 (0.940–0.983)	0.973 (0.948–0.988)	0.970 (0.944–0.986)	0.947 (0.915–0.969)	
Referable GON							
*Model 1*							
AUC	0.675 (0.604–0.746)	0.862 (0.797–0.926)	0.894 (0.836–0.953)	0.976 (0.946–1.000)	0.953 (0.911–0.994)	0.994 (0.988–0.999)	<0.001
Kappa	0.445 (0.341–0.549)	0.779 (0.666–0.891)	0.825 (0.712–0.938)	0.961 (0.848–1.000)	0.922 (0.809–1.00)	0.926 (0.813–1.00)	
Agreement rate	0.887 (0.845–0.920)	0.947 (0.914–0.969)	0.957 (0.927–0.977)	0.990 (0.971–0.998)	0.980 (0.957–0.993)	0.980 (0.956–0.993)	

DR, diabetic retinopathy; AMD, age-related macular degeneration; GON, glaucomatous optic neuropathy; AUC, area under receiver operator characteristic curve; CI, confidence interval. ^a^The AUC, kappa, and agreement rate of graders and ophthalmologists were calculated using a representative grading result for each group when there was at least 50% of group members reached consistent grading.

### Ophthalmologist characteristics related with image interpretation agreement

The agreement between general ophthalmologists’ image grading and the reference standard is shown in Table 4. Table 4 shows that the overall agreement was higher for referable DR in ophthalmologists with greater clinical experience (*p* = 0.009) and those who were specialists (*p* = 0.040). Agreement was significantly higher for referable AMD in ophthalmologists from provincial level hospitals (*p* = 0.017), adjunct academic affiliations (*p* = 0.002), ophthalmologists with more years of clinical practice (*p* = 0.009), and those who were glaucoma or retinal specialist ophthalmologists (*p* = 0.006). Similarly, the level of agreement for referable GON was greater among ophthalmologists from provincial level hospitals (*p* < 0.001), those from adjunct academic affiliations (*p* < 0.001), those with more years of clinical experience (*p* < 0.001) and those who were glaucoma or retinal specialist ophthalmologists (*p* < 0.001).

**Table 4:** Ophthalmologist characteristics for image interpretation versus expert consensus reference standard.

Characteristics	Referable diabetic retinopathy				Referable age-related macular degeneration				Referable glaucomatous optic neuropathy			
	*n*	AUC (95% CI)	Agreement rate (95% CI)	*p*	*n*	AUC (95% CI)	Agreement rate (95% CI)	*p*	*n*	AUC (95% CI)	Agreement rate (95% CI)	*p*
Hospital												
*County level* (*n =* 20)	5,794	0.929 (0.929–0.930)	0.955 (0.955–0.956)		5,878	0.871 (0.871–0.872)	0.929 (0.929 0.930)		5,868	0.818 (0.818–0.820)	0.903 (0.902–0.903)	
*Provincial level* (*n =* 27)	7,894	0.932 (0.931–0.932)	0.956 (0.956–0.957)		7,971	0.872 (0.871–0.872)	0.929 (0.929–0.930)		8,030	0.875 (0.875–0.876)	0.933 (0.932–0.933)	
				0.808^a^				0.017^a^				<0.001^a^
Academic affiliation												
*None* (*n =* 18)	5,196	0.926 (0.925–0.926)	0.954 (0.954–0.955)		5,281	0.867 (0.867–0.868)	0.926 (0.926–0.927)		5,269	0.810 (0.810–0.811)	0.897 (0.897–0.898)	
*Adjunct affiliation* (*n =* 29)	8,492	0.934 (0.934–0.935)	0.957 (0.957–0.958)		8,568	0.891 (0.891–0.892)	0.941 (0.941–0.942)		8,629	0.877 (0.876–0.878)	0.934 (0.934–0.935)	
				0.343^b^				0.002^b^				<0.001^b^
Clinical practice (yrs)												
≤5 (*n =* 13)	3,718	0.925 (0.924–0.925)	0.951 (0.950–0.951)		3,780	0.875 (0.875–0.876)	0.928 (0.927–0.928)		3,782	0.806 (0.805–0.807)	0.569 (0.892–0.893)	
5–10 (*n =* 16)	4,637	0.929 (0.928–0.929)	0.953 (0.953–0.954)		4,703	0.876 (0.876–0.877)	0.934 (0.934–0.935)		4,743	0.848 (0.847–0.849)	0.919 (0.919–0.920)	
>10 (*n =* 18)	5,333	0.937 (0.937–0.938)	0.839 (0.838–0.840)		5,366	0.892 (0.891–0.892)	0.942 (0.942–0.943)		5,373	0.887 (0.886–0.888)	0.941 (0.940–0.041)	
				0.009^c^				0.009^c^				<0.001^c^
Expertise in ophthalmology												
*Nonexpert* (*n =* 27)	7,797	0.929 (0.929–0.930)	0.953 (0.953–0.954)		7,919	0.873 (0.873–0.874)	0.930 (0.930–0.931)		7,934	0.817 (0.816–0.817)	0.902 (0.901–0.902)	
*Expert* (*n =* 20)	5,891	0.933 (0.933–0.934)	0.960 (0.960–0.961)		5,930	0.894 (0.894–0.895)	0.942 (0.942–0.943)		5,964	0.898 (0.898–0.899)	0.944 (0.944–0.945)	
				0.040^d^				0.006^d^				<0.001^d^

a A test for trend based on logistic regression model which diagnostic agreement for corresponding disorder was considered as the outcome variable and a two-category variable for hospital level was regarded as independent variable.

b A test for trend based on logistic regression model which diagnostic agreement for corresponding disorder was considered as the outcome variable and a two-category variable for whether to be an adjunct affiliation was regarded as independent variable.

c A test for trend based on logistic regression model which diagnostic agreement for corresponding disorder was considered as the outcome variable and a three-category variable for clinical practice years was regarded as independent variable.

d A test for trend based on logistic regression model which diagnostic agreement for corresponding disorder was considered as the outcome variable and a two-category variable for expertise in ophthalmology was regarded as independent variable.

CI, confidence interval.

### Image disagreement characteristics

**Figure 2:**
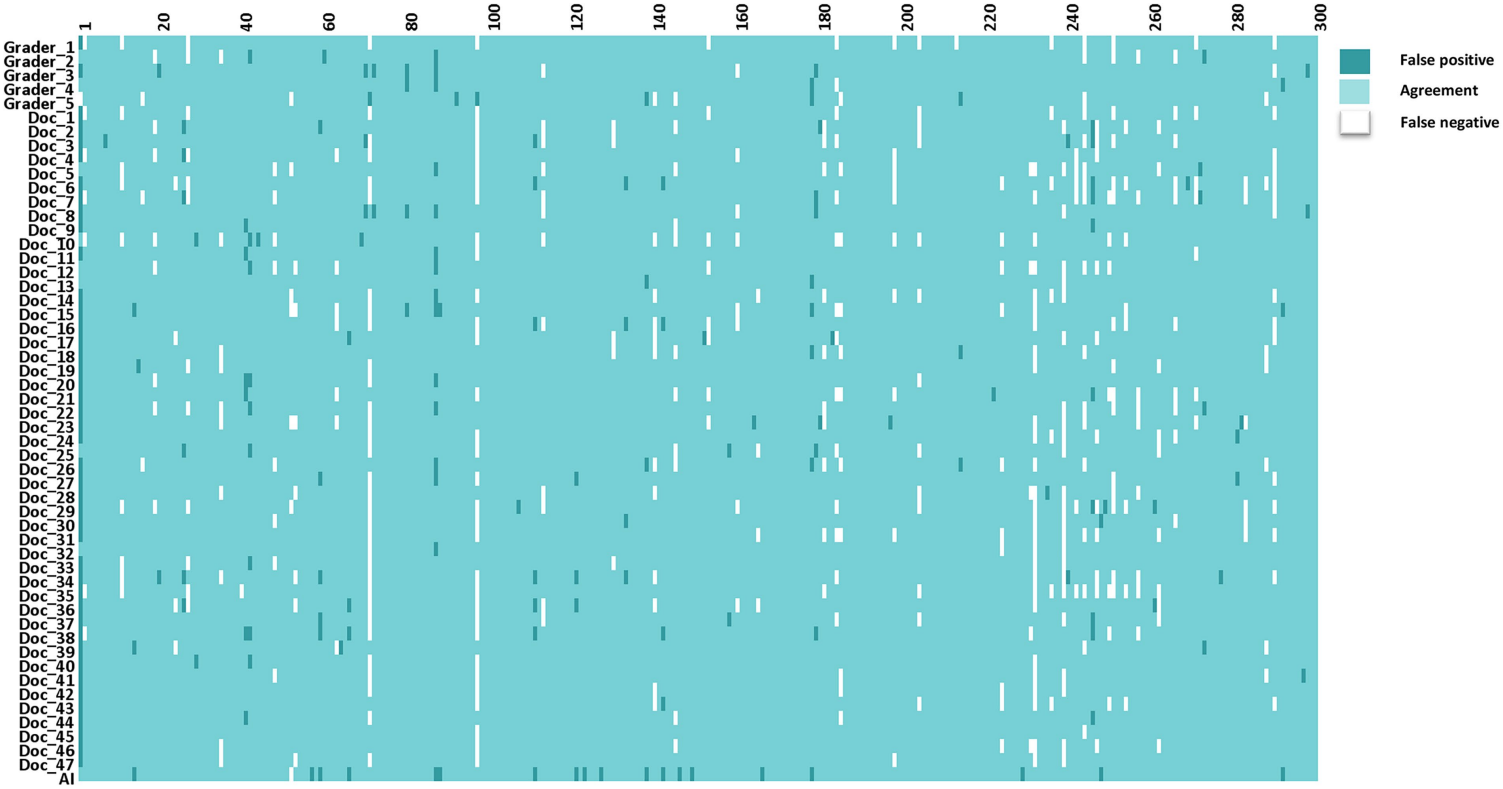
The interpretations of graders, ophthalmologists, and artificial intelligence compared with the reference standards for each of the 300 fundus photographs for diabetic retinopathy.

**Figure 3:**
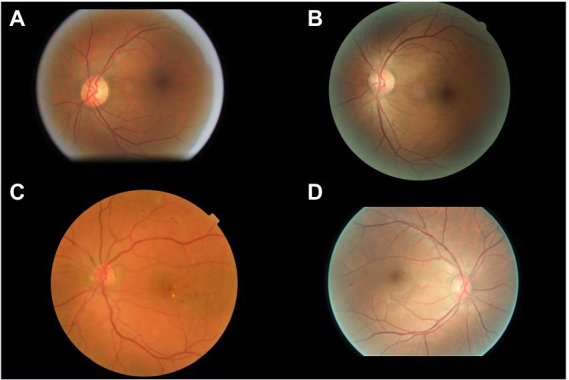
Sample images consistently misclassified by human participants. **(A,B)** Images with only microaneurysm misclassified as referable diabetic retinopathy. **(C)** Images of diabetic macular edema misclassified as non-referable diabetic retinopathy. **(D)** Microaneurysm and dot hemorrhage misclassified as non-referable diabetic retinopathy.

The interpretations of non-physician graders, ophthalmologists, and the DLS compared with the reference standard for each of the 300 fundus photographs for diabetic retinopathy are shown in Figure 2. This figure also demonstrates that several images caused mistakes common to nonphysician graders, ophthalmologists, and the DLS; for example, images #1 and #87 triggered consistent false positives. In the same way, images #71, #97, #140, #181, #232, and #239 displayed consistent false negatives. These images are shown in Figure 3. The general features of images that were misclassified by human participants (trained non-physician graders and ophthalmologists) are summarized in Table 5.The primary reason for false negative of referable DR was the presence of DME (*n* = 10, 58.9%), while two cases (100.0%) with microaneurysm/s and artifacts resulted in false positive by human participants. For referable AMD, false negative cases were mostly related to the presence of subtle subretinal hemorrhage (*n* = 6, 50.0%). False positives resulted from misclassification of earlier forms of AMD (*n* = 9, 75.1%). Among human participants, the most common reason for false negative of referable GON were those images with borderline VCDR (*n* = 8, 27.7%), while false positives occurred in those images which displayed physiological cupping (*n* = 14, 93.3%).

**Table 5:** Characteristics of the disagreement images by human participants.^a^

Reason	No.	Proportion (%)
Referable DR		
False negative		
MA, hemorrhage, DME	10	58.9
Dot hemorrhage, MA	4	23.5
MA, hemorrhage, HEs, CWS	3	17.6
Subtotal	17	100.0
False positive		
Microaneurysm/s, Artifacts	1	100.0
Subtotal	1	100.0
Referable AMD		
False negative		
Subretinal Hemorrhage	6	50.0
Sub-retinal/Sub-RPE fibrovascular proliferation	3	25.0
Serous detachment of the sensory retina or RPE	3	25.0
Sub-total	12	100.0
False positive		
Other macular degeneration	9	75.1
Myopic maculopathy	1	8.3
Choroidal osteoma	1	8.3
Other diseases (Pre-macular hemorrhage)	1	8.3
Sub-total	12	100.0
Referable GON		
False negative		
Borderline VCDR	8	27.7
Borderline VCDR with RNFL defect	6	20.7
Optic disk with tilt or rotation	5	17.2
With other diseases	3	10.3
Rim < 0.1	3	10.3
Notch	2	6.9
Linear hemorrhage around optic disk	2	6.9
Sub-total	29	100.0
False positive		
Physiological large cupping (0.5 ≤ VCDR < 0.7)	14	93.3
Juxtapapillary capillary hemangioma	1	7.7
Sub-total	15	100.0

a The cases included in this analysis were those with more than 20% of the individual human participants (graders and ophthalmologists) inconsistent with the reference standard. DR, diabetic retinopathy; MA, microaneurysm; HEs, hard exudates; CWS, cotton-wool spot; DME, diabetic macular edema; AMD, age-related macular degeneration; RPE, retina pigment epithelium; and GON, glaucomatous optic neuropathy; VCDR, vertical cup to disc ratio; RNFL, retinal nerve fiber layer.

One fundus image demonstrated coexisting intraretinal microvascular abnormality and DME that were not identified by the DLS. The most common reason for false positives by the DLS was the presence of microaneurysm/s only (*n* = 10, 55.5%; Table 6). For referable AMD, the presence of subretinal hemorrhage (*n* = 5, 71.4%) was the primary reason for false negative and other diseases (*n* = 7, 87.5%) including DR or GON. For referable GON, the DLS under-interpreted one image with VCDR less than 0.7, while two images with physiological large cupping (*n* = 2, 40%) and three images with other diseases (*n* = 3, 60%) were incorrectly classified as positive.

**Table 6:** Characteristics of the disagreement images by deep learning system.

Reason	No.	Proportion (%)
Referable DR		
False negative		
MA, IRMA, DME	1	100.0
Sub-total	1	100.0
False positive		
MA only	10	55.5
Other diseases		
Late wet AMD	4	22.2
Retinal degeneration	3	16.7
RVO	1	5.6
Subtotal	18	100.0
Referable AMD		
False negative		
Subretinal hemorrhage	5	71.4
Serous detachment of the sensory retina or RPE	2	28.6
Subtotal	7	100.0
False positive		
Other diseases		
DR	7	87.5
GON	1	12.5
Subtotal	8	100.0
Referable GON		
False negative		
VCDR < 0.7 with notch	1	100.0
Sub-total	1	100.0
False positive		
Physiologic large cupping (0.5 ≤ VCDR < 0.7)	2	40.0
Other diseases		
AMD	2	40.0
Juxtapapillary capillary hemangioma	1	20.0
Subtotal	5	100.0

DR, diabetic retinopathy; MA, microaneurysm; IRMA, intra-retinal microvascular abnormality; DME, diabetic macular edema; AMD, age-related macular degeneration; VRO, retinal vein occlusion; RPE, retina pigment epithelium; and GON, glaucomatous optic neuropathy.

## Discussion

In this study, we prospectively compared the diagnostic agreement of trained non-physician graders and ophthalmologists using three validated deep learning models for the detection of referable DR, late wet AMD, and GON from color fundus photographs. Our results suggest that the performance of the deep learning models for referable DR and AMD are comparable to non-physician graders and ophthalmologists. As for referable GON, the DLS outperformed non-physician graders.

There was no difference among the non-physician graders, ophthalmologists with different years of clinical practice, and the DLS for the diagnostic accuracy of referable DR. The non-physician graders included in this study all had grader certification from the NHS DR screening program, underwent regular assessments every month, and routinely interpreted fundus photographs of diabetic patients from nationwide screening programs, which may explain their relatively high agreement compared to the gold standard. While the DLS also exhibited comparably good performance when compared with non-physician graders and general ophthalmologists.

Comparison of the DLS with general ophthalmologists found that the DLS had higher sensitivity (97.8 vs. 91.1%) and lower specificity (92.5 vs. 99.6%) for the classification of referable DR. However, nearly half of the false positive cases identified by the DLS included (*n* = 8, 44.5%) other disorders, for example, late wet AMD and retinal degeneration. The remaining false positive images (*n* = 10, 55.5%) had mild NPDR. Those images identified as false positive by the DLS would receive a referral and be identified during confirmatory examination conducted by a specialist.

Previous studies have shown that the majority of referral cases for DR (73%) are as a result of DME (35). There are 100 million patients with DR worldwide which corresponds to 7.6 million DME patients (36). However, our results showed that images that were characterized as DME (*n* = 10, 58.9%) were under interpreted by human graders more often than other DR lesions. DR changes related to DME displayed considerable variation among graders and ophthalmologists, with an overall agreement rate of 71% when compared with the reference standard. Therefore, the importance of not overlooking the diagnosis of DME among graders and ophthalmologists should be emphasized.

The DLS outperformed non-physician graders in the classification of referable GON in this study. The variability in inter-assessor agreement among non-physician graders and ophthalmologists for the classification of ocular disorders is well known, especially glaucoma (37, 38). The Glaucomatous optic neuropathy evaluation (GONE) project previously reported that ophthalmology trainees underestimated glaucoma likelihood in 22.1% of optic disks and overestimated 13.0% of included optic disks. This has been similar in our study where general ophthalmologists underestimated 23.8% and underestimated 8.9% of included optic disks (37). Furthermore, Breusegem et al. (38) reported that non-expert ophthalmologists had significantly lower accuracy compared with experts in the diagnosis of glaucoma. Our results are in agreement with previous studies and showed that ophthalmologists with more clinical experience and specialist training in ophthalmology achieve higher inter-assessor agreement. The experience and knowledge obtained through years of clinical practice is likely to play a significant role in interpretation and performance accuracy. In contrast, the DLS is easily able to adopt labels from experienced ophthalmologists to learn the most representative characteristics of GON. Fundus photography is an important method to evaluate GON, however, the diagnosis of glaucoma requires the results of visual field analysis, optical coherence tomography, and intra ocular pressure measurements to make an accurate diagnosis. Thus, further studies to compare DLS with ophthalmologists using multi-modality clinical data is warranted.

The main strength of our study was to prospectively compare the performance of a DLS for the detection of three common blinding eye diseases to non-physician graders and ophthalmologists of varying levels of experience and with different specialties. Our study is also distinctly different from previous reports (19, 39–42). First, we evaluated three ocular diseases at the same time. Second, no prospective comparison of ophthalmologists with varying levels of clinical experience and trained non-physician graders with a DLS for common ocular disorders has been reported. Previous authors have compared the performance of the DLS with that of graders or specialists; this is often considered the gold standard for the development of the DLS (39, 41, 43). Non-physician graders and ophthalmologists are susceptible to making diagnostic mistakes. Our study included independent graders and ophthalmologists to evaluate the performance of the DLS. Therefore, the current study will provide information on the accuracy of the DLS, as well as a more comprehensive understanding and acceptance of how AI systems might work or contribute.

There are several limitations of this study which warrant further consideration. On one hand, human participants included in this study were recruited from China. This has the potential to affect the generalizability of these results to other human graders, especially those in developed countries. In the future, similar studies should be attempted in other countries with different physician or specialist training system. On the other hand, the use of single-field, non-stereoscopic fundus photographs without the inclusion of optical coherence tomography may lead to a reduced sensitivity for DR and particularly DME detection for human participants and the DLS.

In conclusion, our DLS demonstrated sufficient agreement with non-physician graders and general ophthalmologists when compared to the reference standard diagnosis agreement for referable DR and AMD. The DLS performance was better than non-physician graders and ophthalmologists with ≤10 years of clinical experience for referable GON. Further investigation is required to validate the performance in real-world, clinical settings which display the full spectrum and distribution of lesions and manifestations encountered in clinical practice.

## Data availability statement

The original contributions presented in the study are included in the article/Supplementary material, further inquiries can be directed to the corresponding author.

## Ethics statement

The studies involving human participants were reviewed and approved by the Institutional Review Board of the Zhongshan Ophthalmic Center, China. Written informed consent for participation was not required for this study in accordance with the national legislation and the institutional requirements.

## Author contributions

ZL and MH were involved in the concept, design, and development of the deep learning algorithm. ZL, XG, JZ, XL, RC, and MH contributed to the acquisition, analysis, and interpretation of data. ZL wrote the manuscript. All authors revised and edited the manuscript. MH is the guarantor of this work and as such has full access to all the data in the study and takes responsibility for data integrity and the accuracy of the data analysis. All authors contributed to the article and approved the submitted version.

## Funding

This work was supported by National Key R&D Program of China (2018YFC0116500), the Fundamental Research Funds of the State Key Laboratory in Ophthalmology, National Natural Science Foundation of China (81420108008), and Science and Technology Planning Project of Guangdong Province (2013B20400003).

## Conflict of interest

The authors declare that the research was conducted in the absence of any commercial or financial relationships that could be construed as a potential conflict of interest.

## Publisher’s note

All claims expressed in this article are solely those of the authors and do not necessarily represent those of their affiliated organizations, or those of the publisher, the editors and the reviewers. Any product that may be evaluated in this article, or claim that may be made by its manufacturer, is not guaranteed or endorsed by the publisher.

## Footnotes

^1^ http://v.labelme.org

## Abbreviations

DLS: Deep learning systems
DR: Diabetic retinopathy
AMD: Age related macular degeneration
GON: Glaucomatous optic neuropathy
AUC: Area under receiver operator characteristic curve
GONE: Glaucomatous optic neuropathy evaluation
VCDR: Vertical cup to disc ratio
RNFL: Retinal nerve fiber layer
NHS: English national health screening
DESP: Diabetic eye screening program
DME: Diabetic macular edema
